# Interactions of Ligand, Aptamer, and Complementary Oligonucleotide: Studying Impacts of Na^+^ and Mg^2+^ Cations on Sensitive FRET-Based Detection of Aflatoxin B1

**DOI:** 10.3390/molecules30102125

**Published:** 2025-05-11

**Authors:** Alexey V. Samokhvalov, Oksana G. Maksimenko, Sergei A. Eremin, Anatoly V. Zherdev, Boris B. Dzantiev

**Affiliations:** 1 A.N. Bach Institute of Biochemistry, Research Center of Biotechnology, Russian Academy of Sciences, Moscow 119071, Russia; 03alexeysamohvalov09@gmail.com (A.V.S.); zherdev@inbi.ras.ru (A.V.Z.); 2 Institute of Gene Biology, Russian Academy of Sciences, Moscow 119334, Russia; maksog@mail.ru; 3 Faculty of Chemistry, M.V. Lomonosov Moscow State University, Moscow 119991, Russia; saeremin@gmail.com

**Keywords:** aptamers, target-induced strand displacement, complexes of nucleic acids, metal cations, Forster resonance energy transfer, fluorescence polarization, isothermal titration calorimetry, mycotoxins

## Abstract

The effects of magnesium and sodium on the interactions between aptamer, its specific ligand, and short complementary oligonucleotides (cDNAs) differing in affinity of their binding with the aptamer were studied. Aflatoxin B1 (AFB1) and AFB1-binding aptamer were used in the study. Dependencies for the aptamer binding with the fluorophore-labeled AFB1 under varied concentrations of the cations were obtained using fluorescence anisotropy measurements. The increase of the aptamer affinity to AFB1 in the presence of cations was demonstrated using fluorescence anisotropy and isothermal calorimetry. The collected data indicate that 300 mM Mg^2+^ (significantly more than the range commonly used in aptamer sensors) provides the best affinity (16.5 ± 2.2 nM) of the aptamer–AFB1 complexation. Sodium decreases the Mg^2+^-modulated affinity at some Na^+^/Mg^2+^ ratios. The aptamer affinity with cDNAs increases with concentration of cations, but not in the same way as for AFB1. Based on the characterized conditions for bimolecular interactions, the ligand-induced displacement of cDNAs was studied with the registration of the Forster fluorescence energy transfer (FRET). The most sensitive revealing of AFB1 (IC10% 3.2 ± 0.3 nM) in this three-compound FRET system was demonstrated for cDNA having an equilibrium constant of the aptamer binding close to the constant of the aptamer–AFB1 reaction.

## 1. Introduction

Aptamers are bioreceptor molecules of an oligonucleotide nature. Their advantages as analytical reagents include the in vitro selection of new aptamers, low-cost synthesis of known aptamers, minimal batch-to-batch variation, strictly localized modifications, and simple and efficient renaturation [1,2]. A wide variety of aptamer-based assay techniques (colorimetric, electrochemical, fluorescent, and others) have been proposed in recent years [2,3].

**Figure 1:**
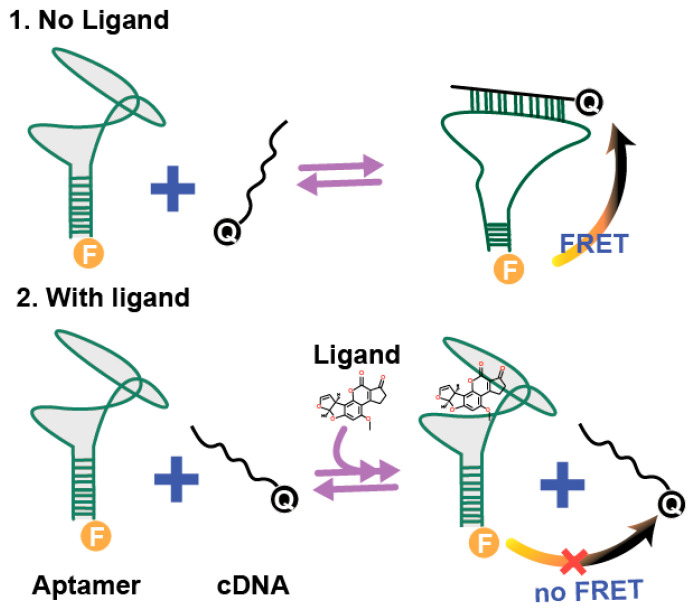
Scheme of the considered aptamer-based assay with ligand-induced strand displacement and Forster resonance energy transfer. F is the donor in the FRET pair and Q is the acceptor.

Aptamer-based assays combining rapidity, low labor-intensiveness, and real-time detection are of great interest [4,5]. The listed features can be reached by ligand (target)-induced strand displacement accomplished with Forster resonance energy transfer (FRET). The principle of this assay (see Figure 1) involves the formation of a duplex between complementary sections of single-strand DNA aptamer and complementary oligonucleotide (cDNA), followed by the dissociation of the duplex caused by the target compound [4]. To achieve FRET, one strand of the duplex is labeled with a donor (fluorophore) and the other strand-with an acceptor (quencher). FRET requires the spectral overlap of the emission spectra of the donor with the excitation spectra, and depends on the distance between them [6]. The formation of the duplex brings the donor and acceptor within an acceptable distance for the FRET (typically several nanometers, depending on the chosen donor–acceptor pair), accompanied by the quenching of the donor’s fluorescence. Adding the ligand to the aptamer–cDNA duplex partially displaces the cDNA from the duplex, thereby increasing the dono–acceptor distance. Thus, the concentration of the ligand in the sample can be determined by the detected donor’s fluorescence [4]. This approach has been successfully implemented for different targets including low molecular compounds (kanamycin [7], ATP [8]), and proteins (nucleolin [9], Ara h1 protein [10]).

Note that new developments are mainly focused on novel aptamer–ligand pairs and new signaling mechanisms for aptasensors [4,11], while little attention is paid to the impact of the composition of the reaction media. To achieve maximum sensitivity in the analytical systems presented above, a rational choice of the reaction medium is needed that integrates different demands for different analytical processes. Under the chosen conditions, the high affinity of the aptamer–ligand binding should be combined with the value of the aptamer–cDNA affinity that provides efficient ligand-induced displacement of cDNA [12,13].

It is known that the analytical characteristics of aptasensors depend significantly on the salt composition of the reaction medium due to the influence of type and concentration of cations on the interactions between complementary bases, both intramolecular (in the aptamer) and intermolecular (in the aptamer and cDNA) interactions [14,15]. The changes in stability of cDNA–aptamer and aptamer–ligand complexes lead to differences in the efficiency of registering complex formation [16].

Seeing the above-mentioned lack of information and the practical demand for it, the studies presented in the article aimed to characterize the influence of metal cations on interactions in an aptamer-based analytical system that integrates ligand-induced displacement of cDNA with fluorescence resonance energy transfer. The differences in the interaction efficiency were considered to choose conditions for the lowest detection limit of the target ligand. First, optimal concentrations of metal cations for aptamer–ligand binding were determined using fluorescence anisotropy (FA) and isothermal titration calorimetry (ITC) techniques. Then, the influence of metal cation concentrations on the aptamer–cDNA binding was studied using the FRET system and the interactions between aptamer and several cDNAs were characterized in terms of equilibrium dissociation constants (K_D_). The data obtained were used to draw conclusions specifying the influence of cation concentrations on interactions in the aptamer-based assay that integrates strand-displacement and FRET processes. Finally, conditions for achieving the minimal detected concentration of a target ligand were proposed.

**Figure 2:**
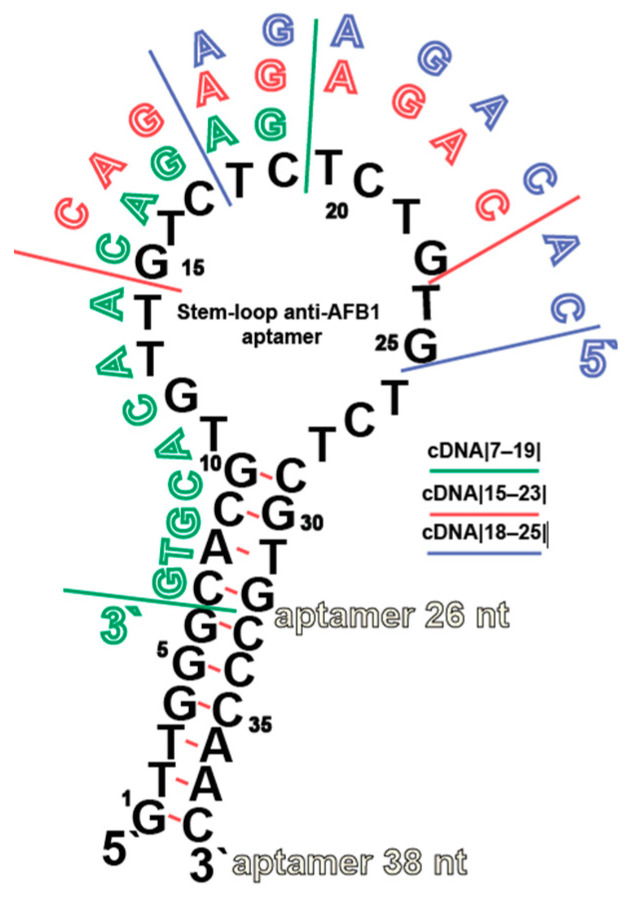
Structures of two variants of AFB1-specific aptamers (38 nt and 26 nt) and three cDNAs. The numbering for the 38 nt goes from the 5′ to 3′-end, and the assigned numbers are used to describe the cDNA located from the 3′ to 5′-end. The cDNAs are specified by hollow letters and marked by green (|7–19|), red (|15–23|), and violet (|18–25|); their correspondence to the aptamer’s nucleotides is specified here and below in the article by ||-marked numbers.

Aflatoxin B1 was chosen as a detected ligand for these investigations. AFB1 is a fungal metabolite, a widespread toxic contaminant of foodstuffs with multiple harmful effects on human health [17]. A Mg^2+^-sensitive anti-AFB1 stem-loop aptamer, initially proposed in [18], was characterized. The strand-displacement processes were carried out through interactions of a fluorophore-labeled aptamer with AFB1 and quencher-labeled cDNA, which are presented in Figure 2.

## 2. Materials and Methods

### 2.1. Reagents and Sample Preparation

The AFB1 powder was obtained from Inikem (Qingdao, China). TAMRA NHS ester(5-isomer) was obtained from Lumiprobe RUS Ltd. (Moscow, Russia). The standard solutions of AFB1, aflatoxin B2 (AFB2), and aflatoxin G1 (AFG1) for HPLC in acetonitrile (10 mg/mL) were from Trilogy (Minot, ND, USA). Synthesis and purification of AFB1 ethylenediamine fluorescein (AFB1-EDF) were performed as described previously [19,20]. The concentration of AFB1-EDF was measured using the calibration curve of fluorescence obtained for known concentrations of fluorescein at excitation wavelength 482 nm and emission wavelength 520 nm.

Tris(hydroxymethyl)aminomethane (99%) and sodium chloride (99.5%) were from Sigma-Aldrich (St. Louis, MO, USA), magnesium acetate (99%) was from Helicon (Moscow, Russia). Lithium acetate (99%) and cesium chloride (99%) was from Vekton (Saint Petersburg, Russia). Potassium chloride (99%) was from Loba Chemie (Mumbai, India). Tween-20 was from Honeywell (Charlotte, NC, USA). All chemicals were analytical grade or chemical reagent grade. A Simplicity Milli-Q^®^ system from Millipore (Darmstadt, Germany) was used to obtain ultrapure water for buffers and solutions of reagents.

All oligonucleotides (see Table 1) and their derivatives labeled with tetramethylrhodamine (TAMRA) or BHQ2 (black hole quencher 2) were custom synthesized and purified by Syntol (Moscow, Russia). Stock solutions of oligonucleotides were prepared by dissolving their lyophilized powder in deionized water. The concentrations of oligonucleotides were measured using a NanoDrop 2000 microvolume spectrophotometer (Thermo Scientific, Waltham, MA, USA), based on optical density at 260 nm.

The interactions were implemented in 20 mM Tris-Acetate buffer, pH 8.5, with 0.02% Tween-20 (hereinafter indicated as TAB) with the addition of NaCl and/or Mg(CH_3_COO)_2_ in different concentrations or without them.

Fluorescence (FL) and fluorescence anisotropy (FA) measurements were performed in black non-binding 96-well NUNC^tm^ microplates from Thermo Scientific (Copenhagen, Denmark), using a CLARIOstar multimode plate reader (BMG Labtech, Ortenberg, Germany). For studies involving fluorescein, the excitation filter 482 ± 16 nm, the dichroic mirror 504 nm, and the emission filter 520 ± 10 nm were used. For studies involving TAMRA the corresponding wavelengths were 540 ± 20 nm, 566 nm, and 590 ± 20 nm, respectively.

### 2.2. The Study of Aptamer–AFB1 Complexation by Fluorescence Anisotropy Measurements Using Fluorescein-Labeled AFB1 Derivative

#### 2.2.1. The Measurements of FA Dependencies of AFB1-EDF on Sodium and Magnesium Concentrations

A series of dilutions of Mg(CH_3_COO)_2_ (from 800 to 5 mM) and NaCl (from 2000 to 8 mM) were prepared in microplate wells. To each well that contains 50 μL of a cation solution the 25 μL of AFB1-EDF (20 nM) in 2x TAB and 25 μL of aptamer (800 nM) in 2x TAB were added sequentially. The microplate was gently shaken for 30 s and incubated at 25 °C for 5 min, after which the FL and FA values were measured. The data were analyzed using CLARIOstar MARS 4.00 R2 software. The ΔFA values were calculated as the differences between FA values of AFB1-EDF with and without the aptamer.

The same protocol was used to obtain the FA dependencies from the sodium/magnesium ratio and study of Mg^2+^ mixes with alkaline metals. Namely, a series of dilutions of NaCl (from 2000 to 8 mM) or cation mixes containing 40 mM Mg(CH_3_COO)_2_ and 400 mM of monovalent cation salt (CH_3_COOLi, NaCl, KCl, or CsCl) were prepared and mixed with AFB1-EDF and aptamer in the proportions mentioned above in TAB. To study the sodium/magnesium ratio TAB contained either 20 or 200 mM of Mg(CH_3_COO)_2_.

#### 2.2.2. The Measurements of Dissociation Constant of Aptamer Interactions with Labeled and Native AFB1 Under Different Conditions Using FA

A series of three-fold dilutions of aptamer 38 nt from 10 to 0.0007 μM were prepared in 50 μL volumes in the wells. To each well 50 μL of 10 nM AFB1-EDF was added and the mixtures were incubated for 5 min at 25 °C before FA measurements. The interactions were performed in TAB with four variants of added salts: 1–300 mM Mg(CH_3_COO)_2_, 2–20 mM Mg(CH_3_COO)_2_, 3–20 mM Mg(CH_3_COO)_2_ with 250 mM NaCl, and 4–1 M NaCl.

After this, a series of three-fold dilutions of AFB1 (from 5000 to 0.25 nM), AFB2, and AFG1 (from 3000 to 0.15 nM) were prepared in microplate wells in 50 μL volumes of deionized water. Then, the equal volumes (25 μL) of aptamer and AFB1-EDF in 2x TAB were added to final concentrations of 90 and 5 nM, respectively. Mixes of AFB1-EDF in 2x TAB with deionized water and 2x TAB or aptamer in 2x TAB were used to obtain FA values of free AFB1-EDF and bound AFB1-EDF, respectively. The prepared mixtures were incubated for 5 min at 25 °C before FA measurements.

The FA values obtained were recalculated to fractions of bound AFB1-EDF (F_bound_) taking into account the change in fluorescence upon binding to the aptamer [21]. Then, the concentration dependencies for the series of experiments described above were plotted in semilogarithmic coordinates and approximated by four-parameter sigmoid fitting with the use of Origin 8.1 software (Origin Lab, Northampton, MA, USA). The values of 50% binding and 50% competition were obtained and used for sequential calculations of the dissociation constant (K_D_) for aptamer interactions with AFB1-EDF and AFB1 by the procedure described in [22,23].

### 2.3. The Measurements of Dissociation Constant of Aptamer–AFB1 Interactions Under Different Salt Conditions Using Isothermal Titration Calorimetry

Isothermal titration calorimetry was performed using the MicroCal PEAQ-ITC titration calorimeter (Malvern Panalytical, Morburn, UK) at 25 °C in TAB, containing 20 or 300 mM of Mg(CH_3_COO)_2_. For the experiments with 20 mM Mg(CH_3_COO)_2_, the concentrations of aptamer and AFB1 were 8 μM and 80 μM, whereas in the case of 300 mM Mg(CH_3_COO)_2_, they were equal to 2 μM and 20 μM, respectively. At first, 280 μL of the aptamer was added into the reaction cell and then the titration syringe was loaded with 40 μL of AFB1. The experiments consisted of 19 successive 2.0 μL injections (except for the first 0.4 μL injection) of AFB1 every 180 s with constant stirring at 750 rpm. To exclude thermal effects associated with dilutions, control experiments with TAB instead of the aptamer solutions were implemented, and the values obtained were used to correct the raw ITC data. The equilibrium dissociation constant (K_D_), stoichiometry (N), enthalpy (∆H), entropy (∆S), and the Gibbs energy (∆G) of the aptamer–AFB1 interactions were determined by the MicroCal PEAQ-ITC Analysis Software v1.41 (Malvern Panalytical, UK) using the one-site binding model.

### 2.4. The Measurements of Fluorescence Resonance Energy Transfer Between Fluorophore-Labeled Aptamer and Quencher-Labeled cDNA

#### 2.4.1. The Study of Duplex Formation of Aptamer with cDNA Under Different Salt Conditions Using FRET Registration

A series of cation concentrations from 800 to 5 mM for Mg^2+^ or from 2000 to 8 mM for Na^+^ were prepared in microplate wells in 50 μL volumes of deionized water. Then 25 μL of 20 nM TAMRA-aptamer in 2x TAB, and 25 μL of cDNAs in 2x TAB or 2x TAB were added to each well. After reagent mixing the microplates were gently shaken for 30 s and after 30 min incubation at 25 °C the fluorescence intensity was measured. The fluorescence values without and with cDNAs at the same salt conditions were measured, the differences between them (∆FL) were calculated, and the obtained dependences were processed using Origin 8.1 software (Origin Lab, USA).

To study aptamer–cDNA complexation, series of cDNAs dilutions from 1600 to 0.1 nM in 50 μL of TAB, containing 100, 40, 20, 10, or 5 mM Mg(CH_3_COO)_2_ or 1 M NaCl, and 7 nM TAMRA-aptamer in TAB with the same salt compositions were prepared. The following incubations and measurements were carried out as for the previous series.

#### 2.4.2. The Study of Competition Between AFB1 and Complementary Strand for Complexation with Aptamer

The successive dilutions of AFB1 were prepared in deionized water. Then, to 50 µL of each dilution, the equal volumes of BHQ2-ssDNA in and TAMRA-aptamer 26nt (14 nM) in 2x TAB with different cation content were added. Wells containing TAMRA-aptamer 26 nt mixed with deionized water and 2x TAB only or with deionized water and BHQ2-ssDNA were used as positive and negative controls, respectively.

Fluorescence measurements were then carried out as described in Section 2.4.1. The data are presented as ∆FL = FL_i_ − FL_negative_, where FL_i_ is the fluorescence at the corresponding AFB1 concentration and FL_negative_ is the fluorescence of the negative control in the absence of AFB1.

## 3. Results and Discussion

### 3.1. The Characteristics of Anti-AFB1 Aptamer

The anti-AFB1 aptamer in question consists of an 18 nucleotide-long loop that provides the AFB1 binding and stem region, providing the loop stabilization. The stem region does not interact with AFB1 and does not affect the affinity of the aptamer to it, but it does stabilize the aptamer [24] and varies in different studies. The stem region should consist of at least 4 base pairs, a shorter length affects aptamer affinity due to the low stability of the structure at 25 °C [24,25]. Two aptamer variants (38 nt and 26 nt) with the same affinity shown using ITC [24] were used in this study to evaluate possible effects of aptamer stability on interactions with cDNAs. The sequences of the both aptamers with labeling sites for FRET measurements are shown in Figure 2, and Table 1.

The aptamer can bind to AFB1 in the absence of cations, but the binding is weak–K_D_ at the 1 μM level [24], which significantly reduces the sensitivity of AFB1 detection. Significant increases in the affinity of the aptamer have been observed in the presence of magnesium in a range from 2 to 100 mM using different methods, including SPR [26], fluorescence anisotropy [20,25], FRET [27], capillary electrophoresis [28], and electrochemical techniques [28]. However, the exact concentrations of Mg^2+^ that provide the minimal dissociation constants (K_D_) of the aptamer–AFB1 complex have not yet been determined.

Sodium (Na^+^) salt is often included in the reaction buffer for the aptamer–AFB1 interaction following the initial SELEX protocol [18]. However, several studies have shown that the presence of Na^+^ is not required for AFB1 binding [26,27].

### 3.2. The Study of Aptamer-Labeled AFB1 Binding on the Concentration of Mg^2+^, Na^+^, and Their Mixtures Using Fluorescence Anisotropy

To obtain FA dependencies of AFB1-EDF binding with the aptamer on concentrations of cations, experiments were carried out under constant aptamer concentration, whereas the concentrations of sodium and magnesium were varied. The aptamer 38 nt, was chosen for the FA experiments, due to its larger size compared to the aptamer 26 nt, resulting in a greater change in the FA of AFB1-EDF after binding.

**Figure 3:**
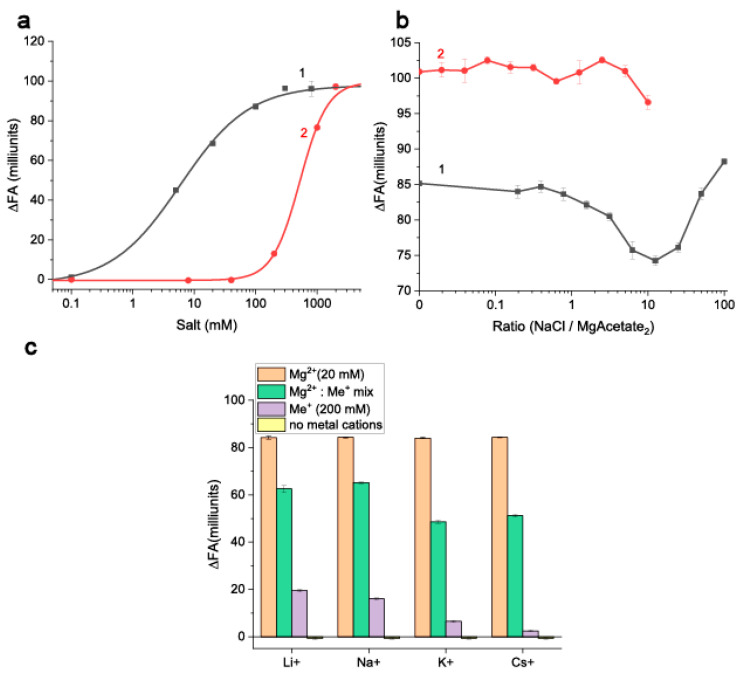
(**a**) The dependencies of the fluorescence anisotropy changes (with 200 nM of aptamer 38 nt vs. without the aptamer) of AFB1-EDF (5 nM) on the concentrations of Mg(CH_3_COO)_2_ (1) and NaCl (2) in TAB. (**b**) The dependencies of the change of fluorescence anisotropy on the NaCl/Mg(CH_3_COO)_2_ ratio in the buffer with 20 (1) or 200 mM (2) of Mg(CH_3_COO)_2_ (n = 2). (**c**) The fluorescence anisotropy changes of AFB1-EDF in the absence and presence of 200 nM of the aptamer 38nt in TAB with different metals cation compositions (n = 3).

The fluorescence of EDF-label without aptamer does not change with the varied concentration of the studied cations, indicating that the cations do not interfere with the measured FA values (Figure S1). The increase in ΔFA (see Figure 3a) indicates an increase in the fraction of AFB1-EDF bound to the aptamer 38nt. As expected, Mg^2+^ modulates the complex formation (Figure 3a (1)). The points of 50% and 95% binding (IC50% and IC95%) of AFB1-EDF were at 6.5 ± 1.0 and 273 ± 52 mM of Mg^2+^, respectively. An interesting fact is that Na^+^ also modulates the formation of the complex, but at significantly higher concentrations–more than 200 mM. The IC50% for Na^+^ was 523.7 ± 32.8 mM. The IC95% for Na^+^ was not reached under the studied conditions and can be estimated to be ≥2400 mM. The observed growth of the aptamer 38 nt affinity with increasing cation concentrations aligns with similar trends that were found for other aptamers with stem-loop structures. For example, the streptavidin-binding aptamer exhibits a three-fold increase in affinity when Na^+^ levels shift from 140 to 350 mM [29]. The IgE-binding aptamer demonstrates a 10-fold increase in affinity when Mg^2+^ concentrations shift from 1 to 40 mM [30]. However, these and other studies demonstrating the same dependencies [31,32,33] were implemented for relatively low or moderate concentrations, not exceeding 400 mM of Me_+_ or 100 mM of Me^2+^. Consequently, the properties of aptamers at higher cation levels remain uncertain. Some studies noted a decrease in aptamer affinity with increasing cation concentrations [34,35]. Therefore, the consideration of novel aptamers should integrate general trends and specific features of their properties in different solutions.

Na^+^ can interfere with the aptamer binding with AFB1-EDF in the presence of Mg^2+^. As follows from Figure 3b, a decrease in ΔFA was observed with increasing Na^+^ in TAB containing 20 mM Mg^2+^. The given decrease reached a maximum at Na^+^: Mg^2+^ molar ratio of 12.5. The increases of Mg^2+^ concentration to 200 mM can fully reduce this effect for all Na^+^ concentrations below 2M.

Earlier studies demonstrated the competition between Mg^2+^ and Na^+^ [36], as well as Mg^2+^ with other cations [37] for nucleic acid binding. Bai et al. [38] found that 88 mM of Na^+^ is needed to displace half of added 5 mM Mg^2+^ from 24 bp dsDNA. This means a 50% exchange requires a 17.6 to 1 Na^+^ to Mg^2+^ molar ratio, which closely matches the maximum decrease in FA of AFB1-EDF. In our case, the obtained data reflect both cation competition and their ability to alter the aptamer structure to enhance ligand binding, making it difficult to estimate cation exchange. Similar effects of other monovalent cations on Mg^2+^-induced binding are expected [37] and shown in Figure 3c. The violet bars demonstrate the increase in modulation of the AFB1-EDF complexation by Me+ in a row Cs+ < K+ < Na+ ≈ Li+, which correlates well with the known affinities of monovalent cations to nucleic acids [37,39]. However, the inhibition of Mg^2+^ -modulated complexation increases in a row Li+ ≈ Na+ < K+ ≈ Cs+ (Figure 3c, green bars). Therefore, the cation that acts as the worst modulator also acts as the best inhibitor, suggesting that more complex processes are involved than just the general differences in nucleic acid binding capability of different cations.

The combination of high affinity and high stability in the presence of other cations makes utilizing high salt conditions perspective, as seen in the example of AFB1. Therefore, using aptamers with optimal affinity at high salt concentrations can be beneficial.

### 3.3. The Measurements of Equilibrium Dissociation Constant Between Aptamer and AFB1-EDF in Reaction Medium with Different Content of Cations Using FA Measurements

Based on the overall assessment of the concentration dependencies presented in Figure 3, we selected four variants of cations contents in the reaction medium (i.e., in TAB):

(A) Mg^2+^ in concentration corresponding to the IC95% in Figure 3 (rounded to 300 mM);
(B) Mg^2+^ in concentration within the generally accepted range (20 mM);
(C) Mg^2+^ and Na^+^ concentrations for the most pronounced effects (250 mM Na^+^ and 20 mM Mg^2+^–ratio 12.5); and
(D) Na^+^ in concentration around the saturation point (1 M).

**Figure 4:**
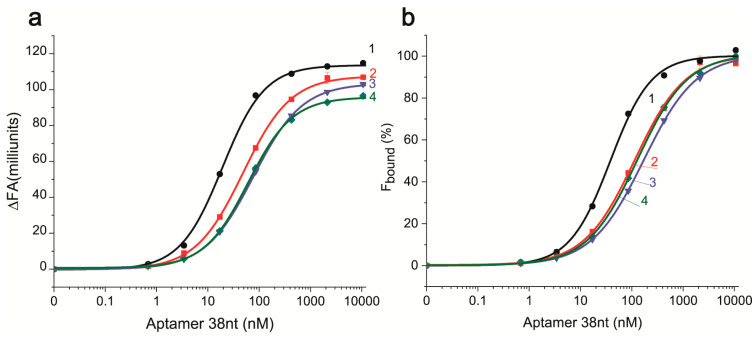
The FA change (**a**) and the percentage of bound AFB1-EDF (**b**) depending on the concentration of aptamer 38 nt in TAB with 1–300 mM Mg(CH_3_COO)_2_, 2–20 mM Mg(CH_3_COO)_2_ 3–20 mM Mg(CH_3_COO)_2_ with 250 mM NaCl, and 4–1 M NaCl (n = 2).

Under these conditions, the dependencies for ΔFA and F_bound_ of AFB1-EDF on the aptamer concentration were obtained; see Figure 4.

The change of aptamer concentration has a different influence on the FA of AFB1-EDF depending on the composition of the reaction media, see Figure 4 (the fluorescence curves presented in Figure S2). By calculating the fraction of bound AFB1-EDF (F_bound_) based of FA measurements (Figure 4b), the equilibrium dissociation constant (K_D_^AFB1-EDF)^) for the aptamer–AFB1-EDF complexes in different media were obtained, see Table 2.

The comparison of obtained constants shows a decrease in the following row of cations in the reaction media: Na^+^/Mg^2+^ ratio 12.5 (0.25 M Na^+^ and 20 mM Mg^2+^) > 1 M Na^+^ > 20 mM Mg^2+^ > 300 mM Mg^2+^. The obtained dependencies can be summarized as the following statements:

- The interference of the aptamer–ligand binding by Na^+^ occurred at certain Na^+^/Mg^2+^ ratios.
- The AFB1-EDF–aptamer binding does require cations and they could be either mono- or divalent.
- The increase of Mg^2+^ up to 300 mM is accompanied with the increase of the K_D_^AFB1-EDF^.

### 3.4. The Measurements of K_D_ for Aptamer–AFB1 Complexes Under Different Concentrations of Magnesium Using FA and ITC

**Figure 5:**
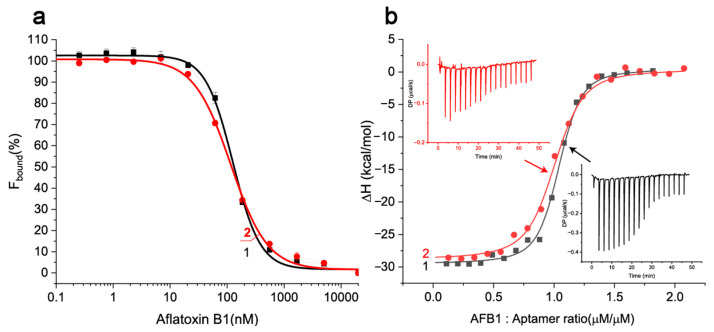
(**a**) The dependencies of percentage of bound AFB1-EDF in presence of 90 nM of aptamer 38 nt from concentration of AFB1 in TAB with (1) 20 mM and (2) 300 mM Mg(CH_3_COO)_2_ obtained by FA measurements (n = 2); (**b**) ITC measurements for sequential additions of AFB1 to aptamer 38 nt and the integrated heat plots for interactions: (1) aptamer (8 μM)–AFB1 (80 μM) in TAB with 20 mM Mg(CH_3_COO)_2_ and (2) aptamer (2 μM)–AFB1 (20 μM) in TAB with 300 mM Mg(CH_3_COO)_2_.

To confirm the increased aptamer affinity for native ligand under high magnesium concentration (300 mM), further studies were conducted using native AFB1. We added native AFB1 as the third interacting compound for studies with FA measurements and obtained ∆FA dependencies from varying concentrations of AFB1, see Figure 5a. In addition, the complexation of native AFB1 and the aptamer 38 nt was characterized in bimolecular system using isothermal titration calorimetry, see Figure 5b. The data from these experiments were processed and the calculated equilibrium binding constants and thermodynamic parameters are summarized in Table 3.

The comparison of FA and ITC data shows that both techniques demonstrated a similar decrease of K_D_ (1.7- and 2.2-fold) with an increase of Mg^2+^ concentration from 20 to 300 mM. The interaction of aptamer 38 nt and AFB1 under both tested Mg^2+^ concentrations was exothermic (∆H < 0) and corresponded to a stoichiometry of 1 to 1.

The collected data indicate that the TAB with 300 mM of Mg^2+^ provided the best affinity of the aptamer–AFB1 complexation. Thus, optimal aptamer–AFB1 binding requires high concentrations of bivalent cations, which differs from the usual recommendations concerning the aptamers use for analytical purposes. The choice of salt composition of the reaction medium appears to be an important starting point for moving towards sensitive aptamer-based analytical techniques.

### 3.5. The Study of Aptamer–cDNA Binding on Concentration of Mg^2+^ and Na^+^ Using FRET

Further estimations of the cation’s effects were carried out for the aptamer–cDNA interactions using TAMRA-BHQ2 as the donor-acceptor pair; see the spectral characterization of these compounds in Figure S3. Earlier studies of this pair demonstrated that the distance between its compounds, at which an 50% efficiency of FRET was achieved (the Förster distance), was between 5 and 7 nm [40,41]. To achieve ~100% efficiency of FRET, the distance should lie within 0.5 of the Förster distance [6].

Two variants of the anti-AFB1 aptamer were labeled by TAMRA and used for FRET studies: (i) the aptamer 38 nt labeled through methyl group of dT28, and (ii) the shorter aptamer 26 nt with TAMRA at the 5′-end. Based on the NMR structure [24], the labelling in both cases should not interfere with AFB1 binding.

**Figure 6:**
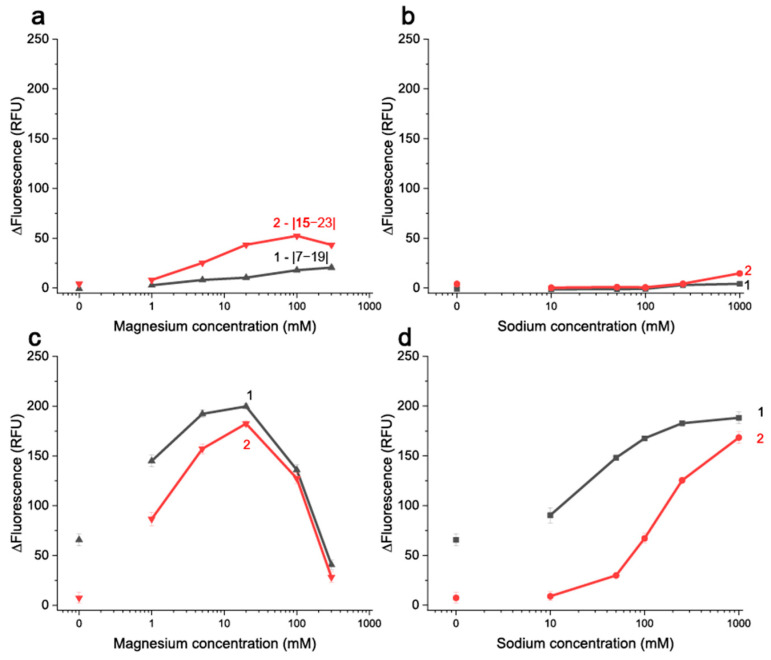
The changes of fluorescence of 10 nM TAMRA-labeled aptamer 38 nt (**a,b**) and aptamer 26 nt (**c,d**) from concentrations of Mg^2+^ (**a**,**c**) and Na^+^ (**b**,**d**) in the presence of (1)—10 nM of BHQ2-labeled |7–19| cDNA and (2)—20 nM BHQ2-labeled |15–23| cDNA (n = 2).

For studies of aptamer–cDNA complexation under different cation content two cDNA, |7–19| and |15–23|, were applied. These cDNAs differ both in location of regions of their binding with the aptamer and the quantity of hydrogen bonds formed as a result of complementary interactions–33 for |7–19| and 24 for |15–23|. The interactions were implemented with constant aptamer: cDNA ratios to obtain the salt conditions providing maximum energy transfer quenching of aptamer in presence of cDNA. The obtained dependencies of ΔFL (differences of FL values without and with cDNAs at the same conditions) from Na^+^ and Mg^2+^ concentrations are given in Figure 6.

For all four tested systems—two aptamers and two cations—the influence of cation’s concentration on the registered ΔFL was observed. The ΔFL increased with the growth of Na+ or Mg^2+^ concentration, causing an increase in the bound fraction of the aptamer as cations tighten the interaction between complementary oligonucleotides. The fluorescence of TAMRA itself is independent of Na+ or Mg^2+^ concentration (Figure S4a). However, in the case of 26 nt in the presence of Mg^2+^, a non-monotonic concentration dependence was observed (Figure 6c). At low concentrations of magnesium (from 1 to 40 mM), the FRET signal increased due to magnesium enhancing the aptamer–cDNA complexation. After reaching a certain concentration point (40 mM), the FRET signal began to decrease due to the loss of free aptamer fluorescence (Figure S4b). TAMRA has a known ability to interact with the bases of oligonucleotides, accompanied by the quenching of its fluorescence through photoinduced electron transfer (PET), especially in complexes with dsDNA [42,43], making it fluorescence sensitive to the folding state of oligonucleotides. To prevent PET, a two-nucleotide linker was introduced between TAMRA and the end of the stem of the 26 nt aptamer. It seems that a high concentration of Mg^2+^ can influence a linker state and put the TAMRA in close proximity to aptamer nucleobases, causing PET-quenching. In the case of TAMRA-aptamer 38 nt, a three-fold decrease in fluorescence was observed (Figure S4c,d) in comparison with free and TAMRA conjugated with 26 nt. It seems that the labeling of dT28, located in the aptamer 38 nt loop leads to the occurrence of PET-induced fluorescence quenching independent from reaction media. The PET interferes the complexation measurements using FRET.

For the accurate data interpretation, it should be noted that a longer stem does not improve affinity, but it does improve the stability of the aptamer and the better stability caused the worse interaction of aptamer with cDNAs [24,25]. Therefore, cDNAs should be chosen for the exact aptamer variant. The advantage of the aptamer 26 nt for further studies of aptamer–cDNA interactions is conditioned by the higher fluorescence of the used label (TAMRA), which provides better sensitivity of complexation detection. The Mg^2+^ concentrations in further experiments were limited to 100 mM to prevent loss of TAMRA fluorescence. The Na^+^ at concentrations exceeding 1 M can also be used to provide effective aptamer binding with cDNA (Figure 6d) as well as ligand (Figure 4a (4)). However, herein, we decide to concentrate on a more commonly used modulator–Mg^2+^.

### 3.6. The Measurements of the Aptamer 26 Nt Interaction with Different cDNAs Using FRET

**Figure 7:**
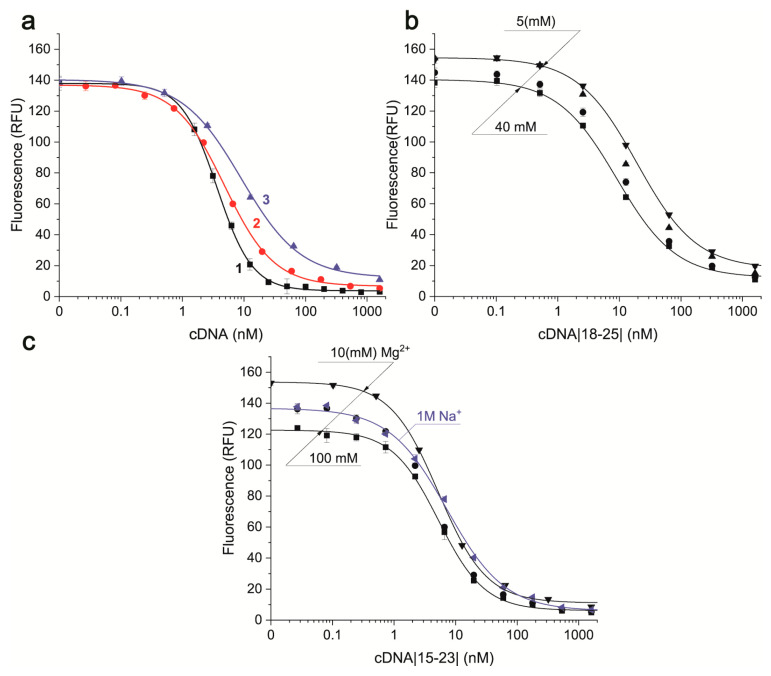
The dependencies of aptamer 26 nt (7 nM) fluorescence on BHQ2-labeled cDNAs concentrations in different reaction media. (**a**) The comparison of cDNAs |7–19| (1), |15–23| (2), and |18–25| (3) in TAB with 40 mM Mg(CH_3_COO)_2_. (**b**) The dependencies for cDNA |18–25| in TAB with different concentrations of Mg^2+^. (**c**) The dependencies for cDNA |15–23| in TAB with different concentrations of Mg^2+^ or with 1 M Na^+^ (n = 3).

The next step was to carry out quantitative characterization of aptamer–cDNA interactions using FRET. The dependencies of fluorescence for TAMRA-labeled aptamer 26 nt on concentrations of cDNAs with different lengths and locations of their complementary regions were presented in Figure 7.

The obtained dependencies were processed using equations for the case when the equilibrium concentrations of both ligand and receptor cannot be omitted [44]. Because the K_D_ values are lower than the label concentration for these conditions, the calculated constants are commonly named “apparent K_D_”. The apparent K_D_ of the reactions between aptamer 26 nt and cDNAs (see Figure 7a) increased with an increase in the number of the formed hydrogen bonds: 0.12 ± 0.07 nM for cDNA |7–19| (33 bonds), 1.33 ± 0.18 nM for cDNA |15–23| (24 bonds), and 5.56 ± 0.78 nM for cDNA |18–25| (20 bonds). These values are about 200-, 20-, and 4-fold higher than the K_D_ of the aptamer–AFB1 reaction under similar conditions, as shown in Table 2.

As shown in Figure 7b, the apparent K_D_ of the aptamer–cDNA reaction increases with the growth of Mg^2+^ concentration in the case of the weakest binding cDNA |18–25|. Namely, changing Mg^2+^ from 5 to 40 mM is accompanied by a three-fold decrease of the apparent K_D_ values from 18.08 ± 0.91 to 5.56 ± 0.78 nM. For the tighter binding cDNA|15–23|, the K_D_ did not change with variation of Mg^2+^ concentration, and only a decrease in ΔFL with increasing Mg^2+^ was observed, see Figure 7c. Apparent K_D_ for cDNA |15–23| vary between 1.3 ± 0.2 and 1.7 ± 0.4 nM, i.e., do not have statistically significant differences. A significant change was observed only with the switch of the stabilizing cation from Mg^2+^ to Na^+^, when the apparent K_D_ increased to 6.00 ± 0.58 nM.

The set of cDNAs with affinities to the aptamer that are comparable or higher by two orders of magnitude than the aptamer–AFB1 affinity was obtained. These allows for the interpretation of the following studies of the combined interactions of these cDNAs and AFB1 with the aptamer.

### 3.7. The Study of Combined Interaction of AFB1 and cDNAs with Different Affinities with the Aptamer

The comparison of cDNAs was implemented under conditions providing a unified fraction of bound aptamer in the absence of AFB1. Namely, the concentrations of cDNAs that caused 70% binding of the aptamer were chosen. As followed from processing the dependencies given in Figure 7, the corresponding IC70% values were 7, 16, and 23 nM for |7–19|, |15–23|, and |18–25| cDNAs, respectively.

**Figure 8:**
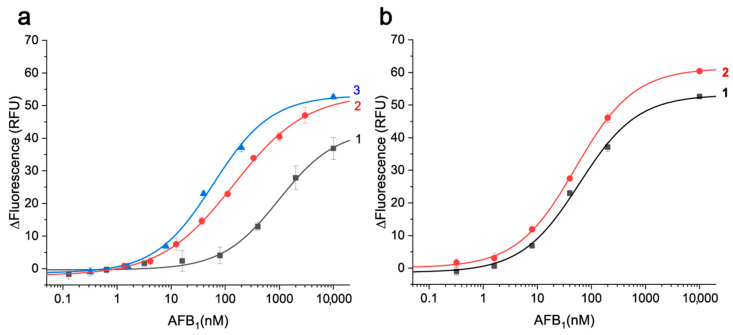
The changes of fluorescence of 7 nM TAMRA-labeled aptamer 26 nt on AFB1 concentration. (**a**) The curves obtained for 7 nM |7–19| cDNA (1), 7 nM |15–23|cDNA (2), and 16 nM |18–25| cDNA (3). The reaction media was TAB with 40 mM Mg^2+^ for all three cases. (**b**) The curves obtained for 16 nM |18–25| cDNA in TAB with 40 mM Mg^2+^ (1) and for 23 nM |18–25| cDNA in TAB with 20 mM Mg^2+^ (2) (n = 3).

The dependencies of fluorescence values on AFB1 concentration obtained for three cDNAs under these conditions are given in Figure 8. Differences between the fluorescence values at plateau (high AFB1 concentrations) and for the free aptamer (without AFB1 and cDNAs) were observed in all cases (see Table S1) reaching 20–30%.

As can be seen from Figure 8a, a more efficient competition resulting in lower AFB1 registration was observed for cDNAs with K_D_ values closer to the K_D_ of the aptamer–AFB1 complexation (curves 2 and 3). The systems with different cDNAs were quantitatively compared using the IC10% calculated from the curves given in Figure 8. The IC10% values reflect the sensitivity of AFB1 detection. When comparing the three cDNAs (Figure 8a), the lowest IC10% was reached for |18–25| cDNA–3.9 ± 1.25 nM, compared to 4.8 ± 1.3 and 68.7 ± 15.8 nM with |15–23| and |7–19| cDNAs, respectively. Additional variation of Mg^2^ concentration used in combination with this optimal cDNA (Figure 8b) demonstrated only a slight decrease in IC10% to 3.2 ± 0.3 nM for 20 mM Mg^2+^.

## 4. Conclusions

The generalization of factors influencing ligand-induced displacement allows proposing the media composition and reagent combination for sensitive ligand’s detection. Several studies have characterized AFB1-induced strand displacements with variations of different parameters (salt concentration, aptamer length, cDNA length, and temperature). As can be seen from earlier and actually obtained data, all parameters affect each other, making the direct comparison of combinations difficult. So, the systems integrating several interactions—such as FRET-based strand displacement–need additional investigation, as well as recommendations for other kinds of sensors cannot be transferred to them.

In our study, the influence of magnesium on the equilibrium in a system involving ligand-induced displacement and fluorescence resonance energy transfer detection was investigated. The concentration of magnesium that provided the best aptamer affinity was determined to be 300 mM, which is 1.7 times better than in the presence of 20 mM magnesium. The ratio of K_D_ between the aptamer–ligand and aptamer–cDNA reactions determines the results of the strand-displacement reaction and each reaction depends on the type and concentration of cations. Too tight cDNA-aptamer binding is undesirable and the K_D_ values should be comparable to obtain the best sensitivity of the ligand detection.

The application of high-salt conditions has the potential to enhance aptamer–ligand interactions and decrease interference of sample composition. These factors can improve the performance of aptamer-based in vitro analytical systems, where the reaction medium is not constrained by physiological conditions or the stability of reagents (labels or target ligands).

## Figures and Tables

**Table 1:** Sequences of the aptamers and the cDNAs used in the study.

Indications	Aptamers (5′-3′)																																						Stem Length	Hydrogen Bonds
	**Stem**										**Loop**																		**Stem**											
	1	2	3	4	5	6	7	8	9	10	11	12	13	14	15	16	17	18	19	20	21	22	23	24	25	26	27	28	29	30	31	32	33	34	35	36	37	38		
38 nt	G	T	T	G	G	G	C	A	C	G	T	G	T	T	G	T	C	T	C	T	C	T	G	T	G	T	C	T	C	G	T	G	C	C	C	T	T	C	10	27
26 nt					A	T	C	A	C	G	T	G	T	T	G	T	C	T	C	T	C	T	G	T	G	T	C	T	C	G	T	G							4	11
**Indications**	**cDNAs (3′-5′)**																																						**Length**	**Hydrogen Bonds**
	1	2	3	4	5	6	7	8	9	10	11	12	13	14	15	16	17	18	19	20	21	22	23	24	25	26	27	28	29	30	31	32	33	34	35	36	37	38		
|7–19|							G	T	G	C	A	C	A	A	C	A	G	A	G																				13	33
|15–26|															C	A	G	A	G	A	G	A	C																9	24
|18–25|																		A	G	A	G	A	C	A	C														8	20

Black The location of BHQ2; Grey the linker between aptamer and TAMRA.

**Table 2:** Equilibrium parameters for the aptamer 38 nt–AFB1-EDF interactions in different media.

Reaction media	Mg(CH_3_COO)_2_ (mM)	NaCl (M)	K_D_^AFB1-EDF^ (nM)
A	300	0	44.9 ± 3.8
B	20	0	106.8 ± 0.8
C	20	0.25	168.8 ± 5.9
D	0	1	122.4 ± 5.1

**Table 3:** Parameters for the aptamer 38 nt–AFB1 interaction in different media.

Fluorescence Anisotropy						
Mg(CH_3_COO)_2_ (mM)	NaCl (M)	L_50_ (nM)				K_D_ (nM)
20	0	126.3 ± 5.8				36.8 ± 2.2
300	0	117.7 ± 6.3				16.5 ± 2.2
**Isothermal Calorimetry**						
**Mg(CH_3_COO)_2_ (mM)**	**NaCl (M)**	**Stoichiometry**	**∆H (kcal/mol)**	**∆S ×** **T (kcal/mol)** **×** **K**	**∆G (kcal/mol)**	**K_D_ (nM)**
20	0	1.00	−29.9 ± 0.4	−19.9	−9.9	42.1 ± 6.2
300	0	0.96	−29.6 ± 0.3	−18.8	−10.4	25.3 ± 3.1

## Data Availability

The data presented in this study are available on request from the corresponding author.

## Supplementary Materials

The following supporting information can be downloaded at: https://www.mdpi.com/article/10.3390/molecules30102125/s1, Figure S1: The dependencies of the fluorescence (ex = 482 ± 16/em = 520 ± 10 nm) of AFB1-EDF (5 nM) on the concentrations of Mg(CH_3_COO)_2_ (1) with 200 nM of aptamer 38 nt or without the aptamer(2) and NaCl (3) with 200 nM of aptamer 38 nt or without the aptamer (4) in TAB (n = 2); Figure S2: The dependencies of AFB1-EDF fluorescence of on the concentration of aptamer 38nt in TAB with 1–300 mM Mg(CH_3_COO)_2_, 2–20 mM Mg(CH_3_COO)_2_ and 3–20 mM Mg(CH_3_COO)_2_ with 250 mM NaCl, and 4–1 M NaCl (n = 2); Figure S3: Overlap between absorption spectrum of BHQ2-cDNA (**1**) and the fluorescence emission spectrum of aptamer-TAMRA (2); Figure S4 (a): The fluorescence of 10 nM TAMRA dye on concentration of Na+ (1) and Mg^2+^ (2) in TAB (n = 2); the fluorescence of 10 nM aptamer 26nt(b) or aptamer 38nt(c) on concentration of Na+ (solid) and Mg^2+^ (dash) in presence of (1)—10 nM of |7–19|, (2)—20 nM |15–23| (2)—without cDNA in TAB (n = 2); (d) The fluorescence intensities of 10 nM of labeled aptamer 38 and 26nt in buffer with 40 mM Mg^2+^; Table S1: The change of aptamer fluorescence in the presence of AFB1 at a plateau at high AFB1 concentrations and the fluorescence of free aptamer in absence of complementary strands.
