# Supplementary material for: Interactions of Ligand, Aptamer, and Complementary Oligonucleotide: Studying Impacts of Na+ and Mg2+ Cations on Sensitive FRET-Based Detection of Aflatoxin B1
Source: Molecules. 2025 May 11;30(10):2125. doi: 10.3390/molecules30102125 (PMC12114067; doi:10.3390/molecules30102125)
Supplement: Supplementary file 1 [file molecules-30-02125-s001.zip › molecules-3589816-supplementary.pdf]

Supplementary Materials

Article

Interactions of Ligand, Aptamer, and Complementary Oligonucleotide: Studying Impacts of Na^+ and Mg^{2+} Cations on Sensitive FRET-Based Detection of Aflatoxin B1

Alexey V. Samokhvalov [1], Oksana G. Maksimenko[2], Sergei A. Eremin[3], Anatoly V. Zherdev [1] and Boris B. Dzantiev [1]*

[1] A.N. Bach Institute of Biochemistry, Research Center of Biotechnology, Russian Academy of Sciences, Moscow 119071, Russia

[2] Institute of Gene Biology, Russian Academy of Sciences, Moscow 119334, Russia

[3] Faculty of Chemistry, M.V. Lomonosov Moscow State University, Moscow 119991, Russia

* Correspondence: dzantiev@inbi.ras.ru; Tel.: +7-495-954-31-42

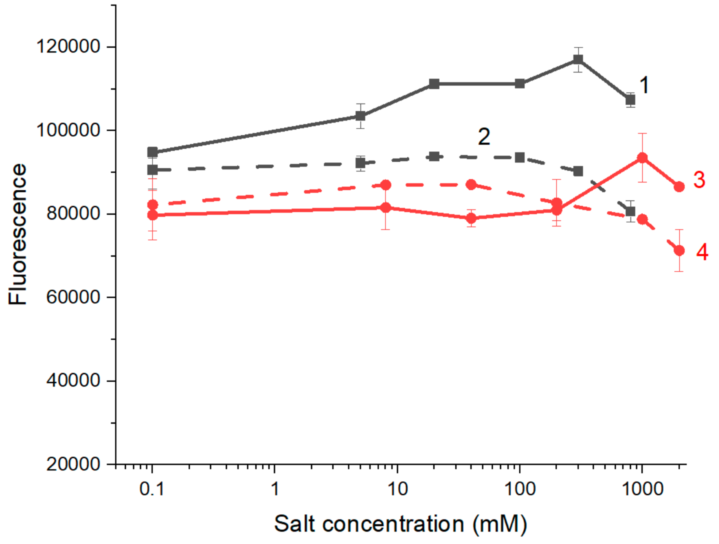

Figure S1. The dependencies of the fluorescence (ex = 482 ± 16 nm / em = 520 ± 10 nm) of AFB1-EDF (5 nM) on the concentrations of $Mg(CH_3COO)_2$ (1) with 200 nM of aptamer 38 nt or without the aptamer (2) and NaCl (3) with 200 nM of aptamer 38 nt or without the aptamer (4) in TAB (n=2).

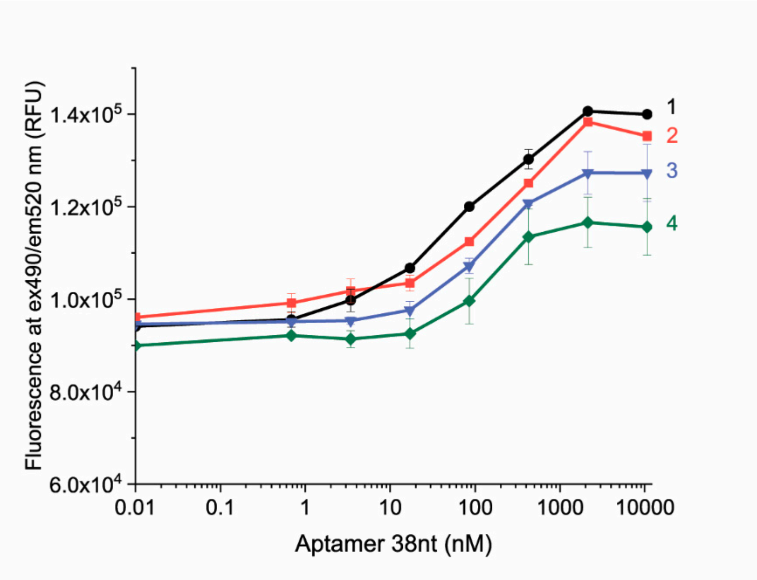

Figure S2. The dependencies of AFB1-EDF fluorescence of on the concentration of aptamer 38 nt in TAB with 1 – 300 mM $Mg(CH_3COO)_2$, 2 – 20 mM $Mg(CH_3COO)_2$ and 3 – 20 mM $Mg(CH_3COO)_2$ with 250 mM NaCl, and 4 – 1 M NaCl (n=2).

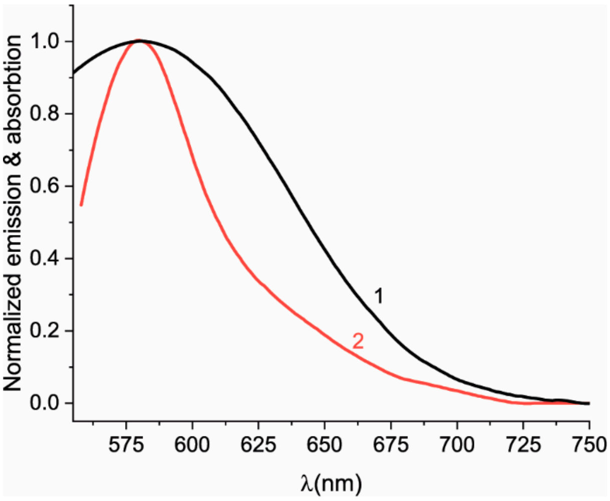

Figure S3. Overlap between absorption spectrum of BHQ2-ssDNA (**1**) and the fluorescence emission of TAMRA-Aptamer (2).

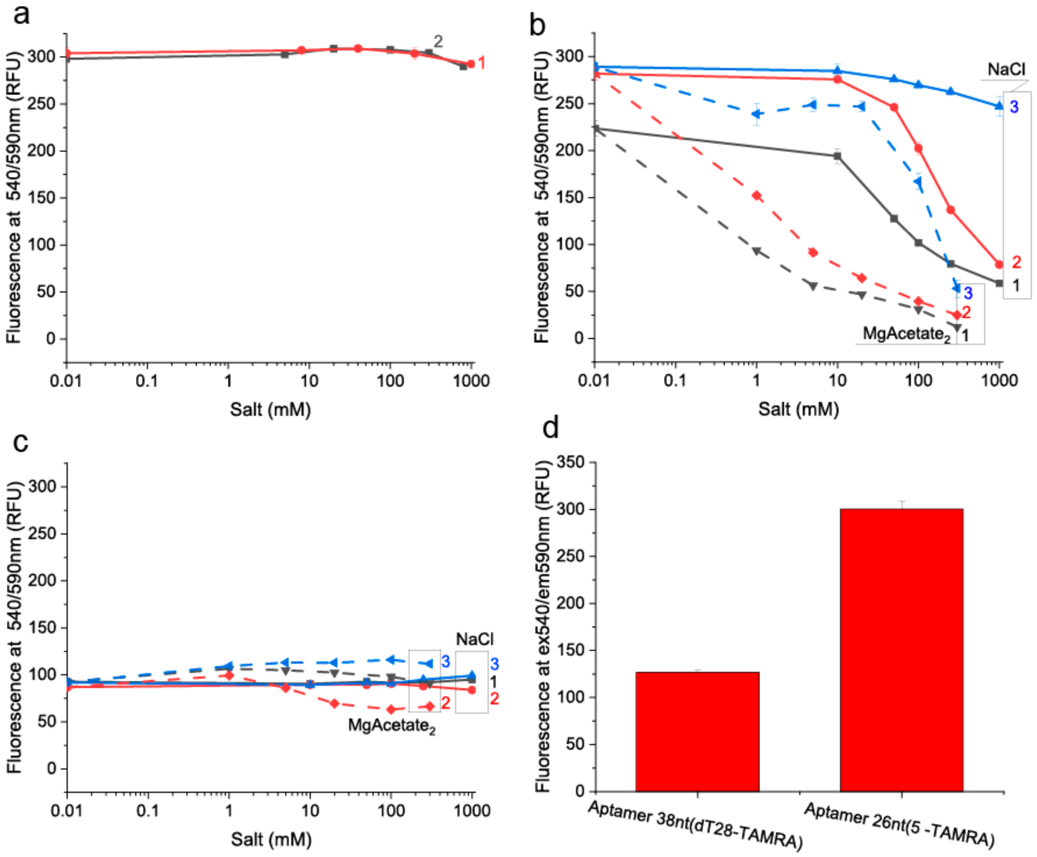

Figure S4. (**a**) – The fluorescence of 10 nM TAMRA dye on concentration of Na^+ (1) and Mg^{2+} (2) in TAB. (n=2). The fluorescence of 10 nM aptamer 26 nt (**b**) or aptamer 38 nt (**c**) on concentration of Na^+ (solid) and Mg^{2+} (dash) in presence of (1) – 10 nM of |7-19|, (2) – 20 nM |15-23| (3) – without cDNA in TAB (n=2). (**d**) – The fluorescence intensities of 10 nM of labeled aptamers 38 nt and 26 nt in buffer with 40 mM Mg^{2+}.

Table S1. The change of aptamer fluorescence in the presence of AFB1 at a plateau at high AFB1 concentrations and the fluorescence of free aptamer in absence of complementary strands

Figure	ssDNA	$Mg(CH_3COO)_2$ (mM)	ΔFluorescence at 10 µM AFB1	ΔFluorescence of aptamer without the cDNA
7a -1	׀7-19 ׀	40	42.62 ± 3.11	90.01 ± 4.69
7a -2	׀15-23׀	40	53.52 ± 2.40	93.24 ± 5.68
7a -3	׀18-25׀	40	53.12 ± 1.68	82.79 ± 8.65
7b	׀18-25׀	20	61.33 ± 0.65	76.789 ± 2.655
